# Editorial: Phenotyping; From Plant, to Data, to Impact and Highlights of the International Plant Phenotyping Symposium - IPPS 2018

**DOI:** 10.3389/fpls.2020.618342

**Published:** 2020-12-04

**Authors:** Cyril Pommier, Trevor Garnett, Carolyn J. Lawrence-Dill, Tony Pridmore, Michelle Watt, Roland Pieruschka, Kioumars Ghamkhar

**Affiliations:** ^1^Université Paris-Saclay, INRAE, URGI, Versailles, France; ^2^Université Paris-Saclay, INRAE, BioinfOmics, Plant Bioinformatics Facility, Versailles, France; ^3^The Plant Accelerator, Australian Plant Phenomics Facility, School of Agriculture, Food and Wine, University of Adelaide, Urrbrae, SA, Australia; ^4^Iowa State University, Ames, IA, United States; ^5^University of Nottingham, Nottingham, United Kingdom; ^6^Faculty of Science, School of BioSciences, University of Melbourne, Parkville, VIC, Australia; ^7^Forschungszentrum Jülich, BG-2: Plant Sciences, Institute for Bio- and Geosciences, Jülich, Germany; ^8^Grasslands Research Centre, AgResearch, Palmerston North, New Zealand

**Keywords:** phenotype, plant science, high throughput phenomics, database (all types), phenomic integration, artificial intelligence, image analysis, data management

The aim of this Research Topic is to provide a series of research articles on a range of subjects in Plant phenomics (Tardieu et al., 2017) from the use of appropriate sensors for capturing morphological and physiological traits to smart ways of processing, extracting and managing “clean” data. Presentation of new approaches to data acquisition, processing and analysis as well as prerequisites for automation are also among the objectives of this Research Topic.

Plant phenomics is the use of sensors, cameras, and algorithms for trait quantification in plants including model species crops, forages, vegetables as well as forest and fruit trees. The relationships between plants and their environment including soil microbes can affect this quantification bringing in new challenging parameters into the equation. Data may be acquired in a range of experimental conditions including laboratories, greenhouse, and field or natural experimental site within forests. Data from the latter can be used for biodiversity studies as well where the scope, measurement means, and objectives can be shifted for that purpose.

This Research Topic presents a series of articles with an insight into recent advances in plant phenomics. There are 12 research articles and one opinion paper covering the heterogeneity and complexity (Watt et al., 2020) of this rapidly developing scientific domain. The majority of the articles address problems associated with data acquisition and analysis using innovative computational methods, and a few discuss artificial intelligence. This reflects the current trend in plant phenomics which leans toward recovering the maximum amount of knowledge and information from the data deluge triggered by high throughput phenotyping. The significance of data management is also highlighted. In this Research Topic, phenomics data acquisition at a very large scale, is demonstrated by Hao et al.. Their work shows how the airborne LiDAR approach allows estimation of the response of forest ecosystems regarding climate change and carbon density. Likewise, Zhang et al. used aerial vectors to enable high throughput data acquisition for understanding leaf development in rapeseed. This approach was taken to obtain usable information for precision farming, including precision fertilization, irrigation, and yield prediction. High throughput phenotyping in automated greenhouse is discussed by Nguyen et al.. They show the production of long time series of images allowing measurement of top view area and shoot biomass to help the estimation of nitrogen use efficiency in wheat.

The imaging types of data produced can also be rather diverse, including color images (as in Hao et al.; Cho et al.; Bateman et al.) but also hyperspectral (Bruning et al.) or Fluorescence (Hupp et al.; Méline et al.) imaging. Méline et al.. suggest promise for estimation of plant response to biotic stress in a non-destructive way in a model plant. This opens up future perspectives for crop research through translational biology.

Major recent innovations in automation and integration of plant phenomics have mobilized the development of deep and machine learning methods and tools and the efforts to address issues with data processing. Dobrescu et al. discuss multitask learning (MTL) to infer two morphological and one classification trait at the same time. Bateman et al. present a new method and name it local context network (LC-Net), which is designed to measure biomass of individual species in a mixed sward using convolutional neural networks.

Atanbori et al. demonstrate the use of conditional Generative Adversarial Network (GAN) to improve the quality of root counting and measurement to train data for missing classes of root images/data.

Semantic approaches are important not only to correctly describe the data but also to extract and organize processed information. Two papers in this Research Topic explore how semantic annotation can be fully or partially automated. Braun and Lawrence-Dill show how *in silico* text mining and natural language processing approaches can be used to extract information from phenotype descriptions, enabling better exploitation of the data gold mine that lies in the literature. Adhikari et al. is more applied and field-based and uses both expert annotation and artificial intelligence approaches to train an automatic weeding method for Paddy fields. Over the past 10 years, scientific communities have proposed several approaches to help data reusability following the FAIR (Wilkinson et al., 2016) principles. This encompasses biologist-friendly data standards such as MIAPPE (Papoutsoglou et al., 2020), ontologies designed to help biologists describe their datasets (Shrestha et al., 2012), or to support data scientists engaged in data integration (Cooper et al., 2017). Technical standards such as the Breeding API (Selby et al., 2019) and databases implementing all of these components such as GnpIS (Pommier et al., 2019) and PHIS (Neveu et al., 2018) have been well-received by the community. In this Topic, MaizeDIG explores the deep integration of phenomic and genomic data using BioDIG (Biological Database of Images and Genomes) web-based software.

The only Opinion paper (Sadras) in this topic gives interesting thoughts on the outcome of the 5th International Plant Phenotyping Symposium (IPPS) 2018, and especially the relationship between theory and our ability to understand and make efficient use of the existing data.

Overall, we believe that the articles published in this Research Topic provide important new knowledge in the broad field of plant phenomics. Our application of sensors, AI, semantics, and data standards and sharing on plant biology is only just beginning. Combining all these tools and techniques to better understand plants and to improve crops are showing immense promise. It is crucial to continue investigating the morphology, physiology, and gene x environment interactions while simultaneously developing and deploying novel approaches to improve the quantification and repeatability of these studies. We hope that the work presented in this Research Topic will help to both consolidate the field of plant-phenotyping and shed light on the significance of methods in decoding plant traits in different environments.

## Author Contributions

All authors listed have made a substantial, direct and intellectual contribution to the work, and approved it for publication.

## Conflict of Interest

The authors declare that the research was conducted in the absence of any commercial or financial relationships that could be construed as a potential conflict of interest.
